# Section of Meteorology, Medical Topography, and Epidemic Diseases

**Published:** 1866-07

**Authors:** 


### SECTION OF METEOROLOGY, MEDICAL TOPOGRAPHY, AND EPIDEMIC DISEASES.

The section was called to order at 3½ o'clock, P.M., May 1st, 1866. Dr. B. H. Catlin, of Conn., was chosen Chairman, and Dr. N. S. Davis, of Ill., Secretary.

A letter from Dr. R. C. Hamil, of Chicago, member of the Committee on Meteorology and Epidemics for the State of Illinois, was presented to the section, stating that important progress had been made in preparing a report, and asking for further time to complete it. On motion of the Secretary, Dr. Hamil was continued a member of the committee another year, and requested to report complete at the next annual meeting of the Association.

The following resolution was offered by the Secretary, and after some remarks on the importance of the several sections of the Association, and the necessity of their perfecting a more permanent and systematic organization, it was adopted as follows:—

*Resolved*, That the section on Meteorology, Medical Topography, and Epidemics, appoint a committee to prepare rules for the permanent organization of said section, with instructions to report at the opening of the section to-morrow.

Drs. N. S. Davis, of Illinois, and B. H. Catlin, of Conn., were appointed said committee.

On motion, the section adjourned to 3 o'clock, P.M., to-morrow.

*Wednesday, May 2d, 1866.*

The section was called to order at 3, P.M., Dr. B. H. Catlin, in the chair. The minutes of the meeting on the previous day were read and approved.

Dr. N. S. Davis, from the committee appointed to report rules for more efficiently organizing the section, reported as follows:—

*By-Laws of the Section of Meteorology, Medical Topography, and Epidemics.*

